# Npp1 prevents external tooth root resorption by regulation of cervical cementum integrity

**DOI:** 10.1038/s41598-022-25846-3

**Published:** 2022-12-07

**Authors:** Hwajung Choi, Liu Yang, Yudong Liu, Ju-Kyung Jeong, Eui-Sic Cho

**Affiliations:** Laboratory for Craniofacial Biology, Cluster for Craniofacial Development and Regeneration Research, Institute of Oral Biosciences, Jeonbuk National University School of Dentistry, 567 Baekje-Daero, Deokjin-Gu, Jeonju, 54896 South Korea

**Keywords:** Developmental biology, Molecular biology

## Abstract

Tooth roots embedded in the alveolar bone do not typically undergo resorption while the bone continues remodeling in its physiological state. In this study, we analyzed genetically modified mice with the functional inactivation of nucleotide pyrophosphatase 1 (Npp1), encoded by ectonucleotide pyrophosphatase/phosphodiesterase 1 (*Enpp1*). This mutation leads to the formation of ectopic cervical cementum vulnerable to external tooth root resorption. Cementoblasts with the inactivation of *Enpp1* extensively expressed non-collagenous matrix proteins enriched with bone sialoprotein (Bsp), dentin matrix protein 1 (Dmp1), and osteopontin (Opn), which have roles in mineralization through nucleation and in cell adhesion through the Arg-Gly-Asp (RGD) motif. In cementoblasts with the inactivation of *Enpp1*, β-catenin was significantly activated and induced the expression of these non-collagenous matrix proteins. In addition, adenosine triphosphate (ATP), which is the most preferred substrate of Npp1, accumulated extracellularly and autocrinally induced the expression of the receptor activator of nuclear factor κB ligand (Rankl) in cementoblasts with inactivated Npp1. Consequently, these results strongly suggest that functional Npp1 preserves cervical cementum integrity and supports the anti-resorptive properties of tooth roots through ATP homeostasis in the physiological state of cervical cementum.

## Introduction

Cementum is a nonuniform mineralized connective tissue covering the tooth root and consists of several types that differ with respect to location, structure, function, rate of formation, chemical composition, and degree of mineralization^1,2^. Overall, cervical acellular cementum forms as a thin solid layer that anchors the periodontal ligament fibers on the growing root surface while apical cellular cementum, as a secondarily formed adaptive tissue, is more bone-like compared with cervical acellular cementum.

At the initiation of physiological external root resorption, the cervical portion of the tooth root is mainly attacked by resorbing cells. For the initiation of resorption, there is some evidence to suggest that the defect of cervical cementum may cause the continued progression of root resorption. The cemento-enamel junction (CEJ) may possess a possible gap between the enamel and cementum and expose underlying the dentin to periodontal ligaments. The CEJ is a main portal of entry for human multiple idiopathic cervical root resorption featured by massive non-inflammatory resorptive lacunae^3,4^. In mouse models, the lack of *Bsp* (*Bsp*^*-/-*^) or the *Col1a1*-conditional deletion of *Alpl* in selected dental cells have the common deficiency at the cervical cementum^5,6^. Considerable destruction of cementum integrity or lack of cervical cementum were followed by further external root resorption or increased numbers of osteoclast-like cells in the tooth of these mice without signs of pathological changes in the periodontium^5,6^. This implies an inborn correlation between cervical cementum integrity with the occurrence of external root resorption.

Cementum has long been regarded as a biomechanically anti-resorptive barrier of tooth roots because it lacks or exhibits minimal remodeling processes of the tissue^7–9^. The cementum matrix contains various extracellular matrix (ECM) proteins that are common to those in the bone, but are less readily resorbed than bone^8^. However, it still remains unclear by which mechanisms attribute to the anti-resorptive properties of the tooth root when compared with bone^9^.

Nucleotide pyrophosphatase 1 (Npp1; encoded by the ectonucleotide pyrophosphatase/phosphodiesterase 1 (*Enpp1*) gene) is a type II extracellular membrane bound glycoprotein and preferentially expressed on the cementoblasts of cervical cementum in tooth roots^10^. As one of the primary enzymes responsible for adenosine triphosphate (ATP) hydrolysis, Npp1 catabolizes extracellular ATP into adenosine monophosphate (AMP) and inorganic pyrophosphate (PPi). As reported previously that loss-of-function mutations in *Enpp1* gene feature a hypercementosis in mice and human disease of generalized arterial calcification of infancy (GACI), PPi functions as a potent inhibitor of ectopic tissue mineralization by binding to nascent hydroxyapatite (HA) crystals^11,12^, thereby preventing the future growth of these crystals in cervical cementum^10^. Though previous studies about Npp1 mainly focused on the mineralization issues with its PPi-generating properties in cementogenesis, understanding the impact of Npp1 on ATP homeostasis affecting ECM regulation for cementum integrity within the larger framework of membrane-associated ecto-nucleotidases is another important issue.

In this study, using genetically modified mice with functional inactivation of *Enpp1* forming ectopic cervical cementum vulnerable to external tooth root resorption, we demonstrated that Npp1 has a role in cementum homeostasis to preserve cervical cementum integrity to protect the tooth root against resorption. Our observations provide a new insight into a novel therapeutic approach for periodontal reconstruction.

## Results

### Functional inactivation of *Enpp1* leads to the formation of cervical cementum vulnerable to resorption

**Figure 1:**
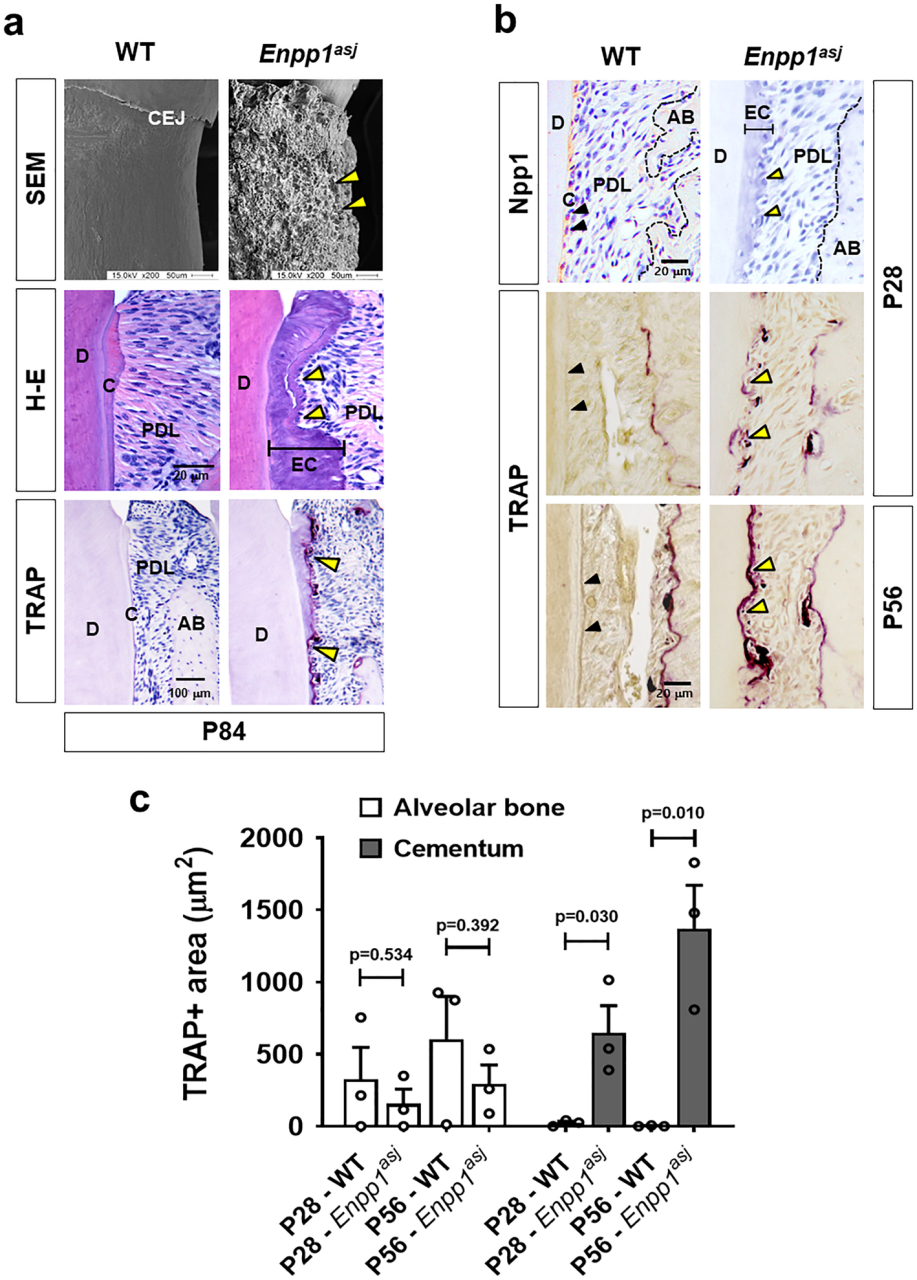
Functional inactivation of *Enpp1* leads to the formation of cervical cementum vulnerable to resorption. (**a**) The surface structure of the distal root of the mandibular first molar at P84 through SEM analysis (Top). Tissue sections of the mandibular first molar from *Enpp1*^*asj*^ and the control mice at P84 were stained with H-E (Middle). TRAP staining was performed with tissue sections of the mandibular first molar at P84 (Bottom). The images of cervical cementum were taken within a site 500 μm apical (one third of total distal root length) from the cemento–enamel junction (CEJ) of the mandibular first molar. (**b**) Molecular changes of Npp1 in the cervical cementum were detected by IHC staining with tissue sections of the mandibular first molar at P28 (Top). The TRAP-positive area of cervical cementum and faced alveolar bone were exhibited with tissue sections of the mandibular first molar at P28 (Middle) and P56 (Bottom). Black arrow heads indicate Npp1-positive and TRAP-negative cervical cementum of WT. Yellow arrow heads indicate resorption lacunae of the thickened cervical cementum layer of *Enpp1*^*asj*^ mice. CEJ, cemento-enamel junction; D, dentin; PDL, periodontal ligament; C, cementum; EC, ectopic cementum; AB, alveolar bone. Scale bars are indicated. (**c**) The TRAP-positive area of cervical cementum and faced alveolar bone were analyzed using tissue sections at P28 and P56 after TRAP staining (n = 3). Significance was assigned with *p*-values as indicated in the graph.

Ectopic cervical cementum formation is a representative alteration caused by matrix apposition and HA crystal formation on cervical cementum by disrupted Npp1 function in the tooth of *Enpp1*^*asj*^^13,14^. Interestingly, the resorption lacuna of thickened cementum was obviously formed at the cervical surface of the tooth root at postnatal day 84 (P84) in *Enpp1*^*asj*^ mice whereas that of wild type (WT) stayed thin and possessed a smooth surface (Fig. 1a). As seen in scanning electron microscopy (SEM) images, the resorption partially extended to the edge of dentin resulting in a very rough surface of cementum with aging while no sign of root resorption on cervical cementum of WT rendering smooth surface. At the younger age of P28, the resorption of thickened cementum in *Enpp1*^*asj*^ mice was already progressed at the cervical aspect of the tooth root as analyzed by TRAP staining while this was not observed in WT (Fig. 1b). In *Enpp1*^*asj*^ mice, most of the lacunae were found preferentially on the distal root surface of molars and cementum-faced mesial sides of alveolar bone that are exposed to low but continuous compressive strains due to physiological distal drift of mouse molars (Supplementary Fig. S1)^15^. Cementum-faced alveolar bones stained with TRAP, especially in the mesial side of alveolar bone between first and second molars, appeared under active remodeling in both mice of P28 and P56. There is no significant difference in TRAP-positive area of alveolar bone adjacent to the resorption lacunae between *Enpp1*^*asj*^ and WT mice while that of cervical cementum has obvious difference (Figs. 1b, c). The severity of resorption in cervical cementum increased with age as indicated by the TRAP-positive area (Fig. 1c). These results imply that Npp1 expressed at the cervical cementum may possess a role in the protection of tooth roots against external resorption.

### Thickened cervical cementum formed by the inactivation of *Enpp1* is enriched with non-collagenous proteins

**Figure 2:**
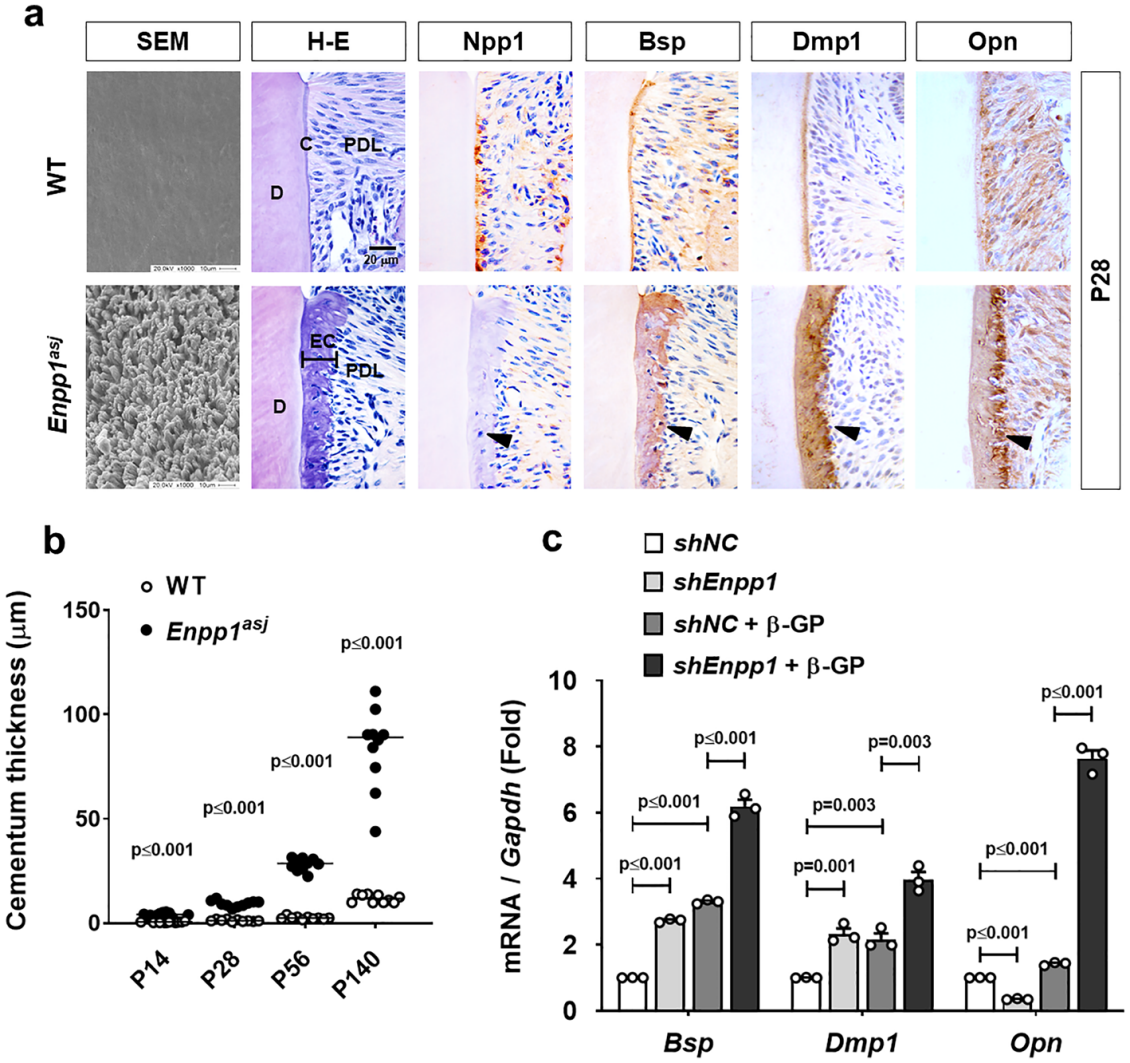
Thickened cervical cementum formed by the inactivation of *Enpp1* is enriched with non-collagenous proteins. (**a**) The surface structure of the distal root of the mandibular first molar at P28 was observed by SEM analysis (Most left). Tissue sections of the mandibular first molar from *Enpp1*^*asj*^ and the control mice at P28 were stained with H-E (Second left). Ectopic cementum (EC) was intensively stained with hematoxylin. Molecular changes of Npp1, Bsp, Dmp1, and Opn in the cervical cementum were detected by IHC staining with tissue sections of the mandibular first molar at P28 as indicated. Black arrow indicates the thickened cervical cementum of *Enpp1*^*asj*^ mice with the IHC pattern that was Npp1-negative and strongly positive with Bsp, Dmp1, and Opn. D, dentin; PDL, periodontal ligament; C, cementum; EC, ectopic cementum. Scale bars are indicated. (**b**) Cementum thickness was measured using H-E-stained tissue sections from WT and *Enpp1*^*asj*^ mice at indicated ages (n = 10). (**c**) Transcript levels of *Bsp*, *Dmp1*, and *Opn* were analyzed by real-time qPCR (n = 3). RNA was isolated from OCCM-30 cells with *shEnpp1* and *shNC* harvested before and after differentiation with 10 mM β-GP treatment for 4 days. Significance was assigned with *p*-values as indicated in the graph.

Since ectopic cervical cementum formed by disrupted Npp1 function in the tooth of *Enpp1*^*asj*^ mice seems to be easily resorbed in vivo, we speculate that this can be attributed to the difference in composition of the cementum matrix when compared with that of WT. SEM images revealed that the surface structure of cervical ectopic cementum in *Enpp1*^*asj*^ mice has a unique cone-shaped structure, implying the rapid deposition of the matrix (Fig. 2a). As seen in hematoxylin–eosin (H–E) staining, the ectopic cementum of *Enpp1*^*asj*^ mice exhibited intense hematoxylin staining. In contrast to a predominant collagenous matrix mainly stained by pinky eosin with H-E staining, non-collagenous proteins are acidic and mainly stained by purple hematoxylin^16^. We speculated that these non-collagenous proteins may play some roles in the resorption process in the ectopic cervical cementum of *Enpp1*^*asj*^ mice. As examined by immunohistochemical staining, thickened cervical cementum is highly enriched with non-collagenous proteins such as bone sialoprotein (Bsp), dentin matrix protein 1 (Dmp1), and osteopontin (Opn) (Fig. 2a), which are known as resorption-susceptible ECM proteins with RGD cell-binding sequence in bone^17,18^. Although the cervical cementum of both WT and *Enpp1*^*asj*^ mice thickened with age, the regional difference in the thickness of cervical cementum was largely increased in *Enpp1*^*asj*^ mice despite partial resorption (Fig. 2b). OCCM-30 cells displaying the stable knockdown of *Enpp1* with small hairpin RNA (shRNA)^14^ also exhibited highly increased expressions of *Bsp*, *Dmp1*, and *Opn* when the cells were differentiated with β-glycerol phosphate (β-GP), an inorganic phosphate, compared to the control (Fig. 2c). Mineralization ability assumed by alkaline phosphatase (ALP) activity and Alizarin red staining was significantly increased in *Enpp1*-knockdown OCCM-30 cells (*shEnpp1*), indicating that Npp1 inhibits mineralization of cementoblasts in vitro (Supplementary Fig. S2).

### Npp1 suppresses β-catenin activity that drives the expression of non-collagenous proteins in Npp1-inactivated cementoblasts

**Figure 3:**
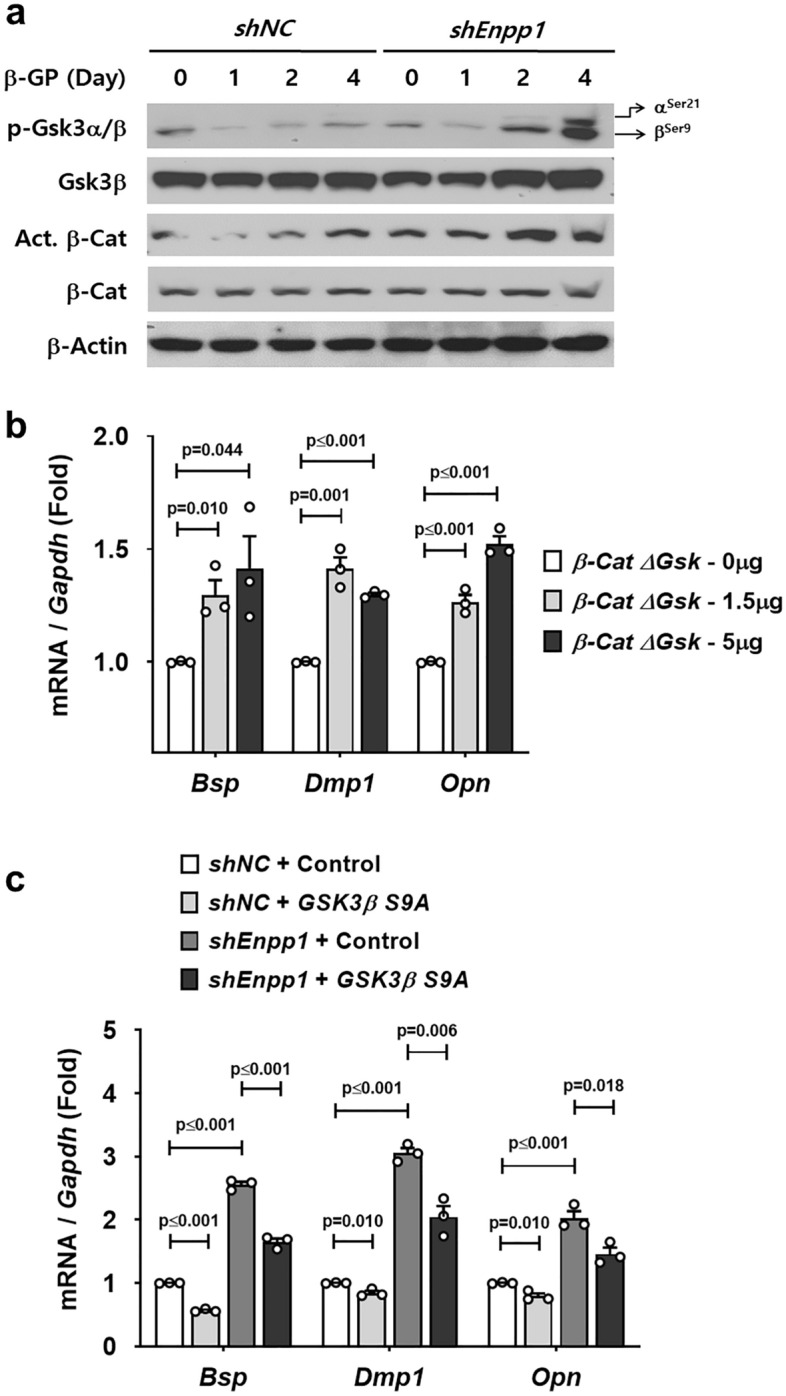
Npp1 suppresses β-catenin activity that drives the expression of non-collagenous proteins in Npp1-inactivated cementoblasts. (**a**) Protein levels were analyzed by Western blotting using specific antibodies with whole cell lysates from OCCM-30 cells with *shEnpp1* and *shNC* harvested after differentiation with 10 mM β-GP treatment for indicated times. Samples shown in Western blotting are from the same experiment, and the gels/blots were processed under the same experimental conditions. β-Actin was used as a loading control. Cropped images are displayed here; the original full-size blots are presented in Supplementary Fig. S5 online. (**b**) The transcript levels of *Bsp*, *Dmp1*, and *Opn* were analyzed by real-time qPCR (n = 3). RNA was isolated from OCCM-30 cells transfected with mouse *β-CatΔGsk* as indicated and harvested after differentiation with 10 mM β-GP treatment for 4 days. (**c**) The transcript levels of *Bsp*, *Dmp1*, and *Opn* were analyzed by real-time qPCR (n = 3). RNA was isolated from OCCM-30 cells with *shEnpp1* and *shNC* harvested after transfection with human *GSK3β S9A* (5 μg) for 24 h and differentiation with 10 mM β-GP treatment for 4 days. Significance was assigned with *p*-values as indicated in the graph.

As previously reported, an excessive cementum matrix is formed on the surface of tooth roots through the stabilization of β-catenin in *osteocalcin* (*OC*)*-Cre:Catnb*^*lox/*+^ mice^19^. This stabilization is achieved through the elimination of the entire exon 3 sequence, which encodes glycogen synthase kinase 3β (Gsk3β) phosphorylation targets in osteocalcin-expressing dental mesenchyme. As analyzed by Western blot, the protein amount of active β-catenin (non-phosphorylated Ser33/37/Thr41) in *Enpp1*-knockdown OCCM-30 cells (*shEnpp1*) gradually increased as differentiation proceeded by β-GP (Fig. 3a). This upregulation of active β-catenin was positively correlated with the amount of phosphorylated Gsk3β (p-Gsk3β), an inactive form of Gsk3β, resulting in the stabilization of β-catenin. However, the total amounts of β-catenin and Gsk3β were not altered with differentiation. To determine whether stabilized β-catenin could drive the secretion of non-collagenous matrix proteins, we analyzed the expression of the non-collagenous matrix proteins after transfection with an active form of mouse *β-catenin*, *β-CatΔGsk* in OCCM-30 cells^20^. The transcripts of all non-collagenous matrix genes tested were upregulated by *β-CatΔGsk* in a concentration-dependent manner as differentiation proceeded by β-GP in OCCM-30 cells (Fig. 3b). Based on this finding, we transfected human *GSK3β S9A*, a constitutively active form of GSK3β, to inactivate endogenous β-catenin in *Enpp1*-knockdown OCCM-30 cells during differentiation. The transcripts of all non-collagenous matrix genes tested were significantly downregulated by *GSK3β S9A* in *Enpp1*-knockdown OCCM-30 cells as well as in the control cells (Fig. 3c). These results suggest that the induction of non-collagenous matrix proteins in ectopic cervical cementum of *Enpp1*^*asj*^ mice is, at least in part, dependent on β-catenin stabilization through the modulation of Gsk3β.

### Inactivation of *Enpp1* induces Rankl expression in cementoblasts

**Figure 4:**
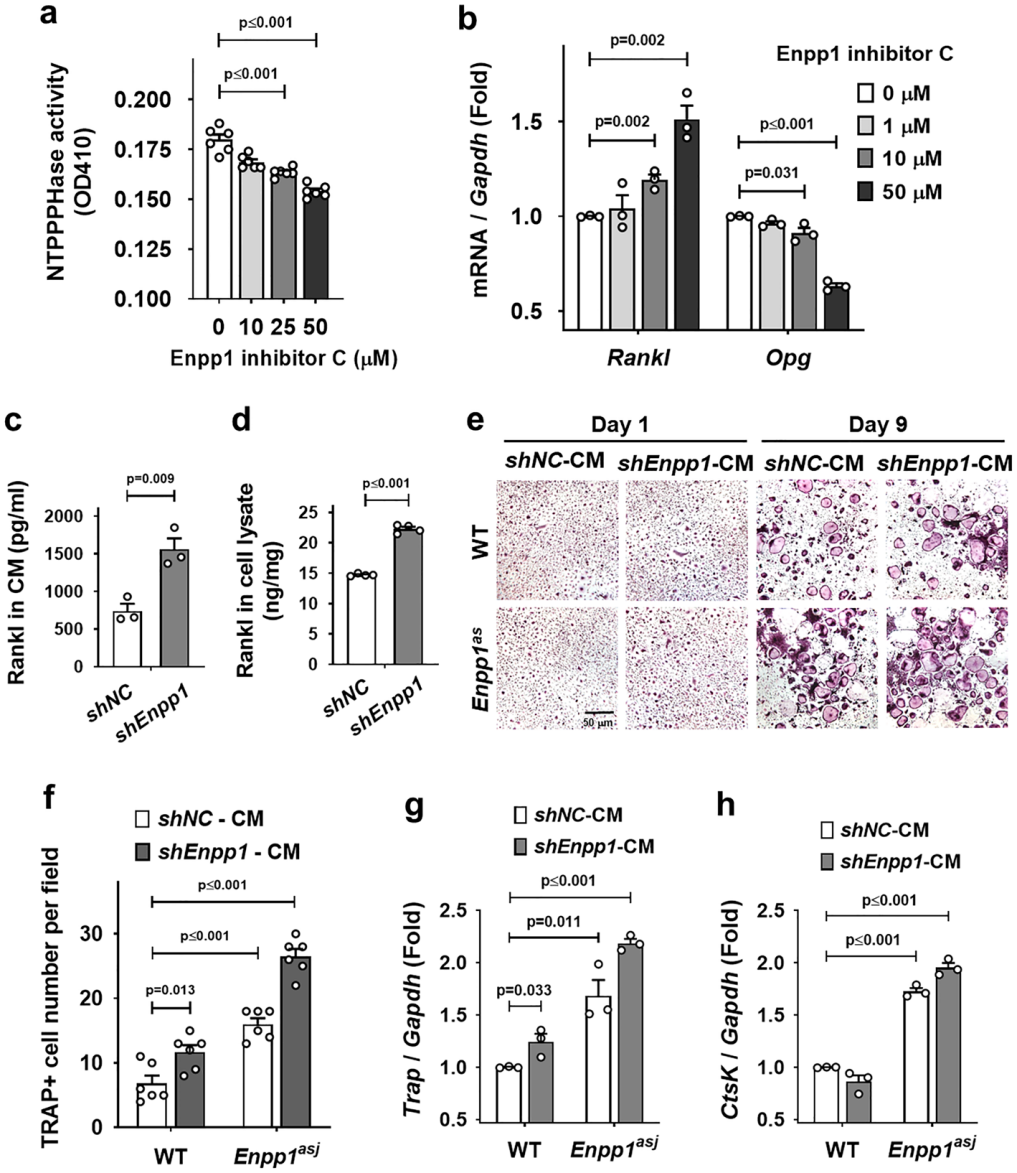
Inactivation of *Enpp1* induces Rankl expression in cementoblasts. (**a**) NTPPPHase activity was analyzed with OCCM-30 cells after treatment with Enpp1 inhibitor C for 2 h as indicated (n = 6). (**b**) Transcript levels of *Rankl* and *Opg* were analyzed by real-time qPCR (n = 3). RNA was isolated from OCCM-30 cells treated with Enpp1 inhibitor C for 24 h. (**c**) The protein amount of soluble Rankl was measured by ELISA using conditioned media (CM) from the culture of OCCM-30 cells with *shEnpp1* and *shNC* (n = 3). (**d**) The protein amount of Rankl was measured by ELISA using whole cell lysates from OCCM-30 cells with *shEnpp1* and *shNC* (n = 4). (**e**) TRAP staining was performed with BMMs from WT and *Enpp1*^*asj*^ mice. BMMs were differentiated for 9 days with conditioned media (CM) from the culture of OCCM-30 cells supplemented with M-CSF (20 ng/ml) but without recombinant Rankl for 9 days. Scale bar are indicated. (**f**) TRAP-positive multinucleated cells were randomly counted in the field of the microscope of (**e**) at day 9 (n = 6). (**g**, **h**) Transcript levels of *Trap* and *CtsK* were analyzed by real-time qPCR (n = 3). RNA was isolated from BMMs differentiated in the same condition with (**f**). Significance was assigned with *p* values as indicated in each graph.

Since ectopic cementum formed by *Enpp1* inactivation is finally resorbed in vivo, we speculated on the presence of permissive levels of Rankl in the cementoblasts of *Enpp1*^*asj*^ mice for macrophages to differentiate into functional resorbing osteoclasts. To verify that Npp1 activity is associated with Rankl expression in cementoblasts, we treated OCCM-30 cells with Enpp1 inhibitor C, a chemical Npp1 inhibitor, and confirmed its concentration-dependent inhibition by an assay for PPi-generating ectoenzyme (nucleoside triphosphate pyrophosphohydrolase, NTPPPHase) activity (Fig. 4a). As expected, the expression of *Rankl* was increased by Npp1 inactivation in a concentration-dependent manner while the expression of *Opg* was decreased (Fig. 4b). These results suggest that the ratio of Rankl/Opg is effectively increased by Npp1 inhibition in cementoblasts. Moreover, as analyzed by ELISA, the expression of Rankl was significantly increased in *Enpp1*-inactivated cementoblasts at both protein levels released into conditioned medium (CM) as well as cell lysate (Figs. 4c, d). To determine whether the soluble Rankl induced in *Enpp1*-inactivated cementoblasts is functional, the CM harvested from the culture of *Enpp1*-knockdown OCCM-30 cells was introduced to bone marrow-derived macrophages (BMMs) without recombinant Rankl during differentiation into multinucleated osteoclasts. BMMs from *Enpp1*^*asj*^ mice were also analyzed in parallel from WT (Fig. 4e). Interestingly, BMMs from *Enpp1*^*asj*^ mice formed higher numbers of TRAP-positive multinucleated osteoclasts after differentiation for 9 days and consequently expressed higher levels of osteoclastogenesis-associated genes such as *Trap* and *cathepsin K (CtsK)* compared to the WT (Fig. 4f–h). The combination of BMMs from *Enpp1*^*asj*^ mice with CM from the culture of *shEnpp1* exhibited the highest osteoclast formation and expression of genes for osteoclastic enzymes after differentiation for 9 days (Fig. 4f–h). These results suggest that the resorption in the cervical cementum of *Enpp1*^*asj*^ mice is driven by both ways from the higher Rankl expression of cementoblasts as well as the intrinsically higher osteoclastic enzyme activity of resorbing cells.

### Extracellular ATP induces the expression of Rankl in cementoblasts

**Figure 5:**
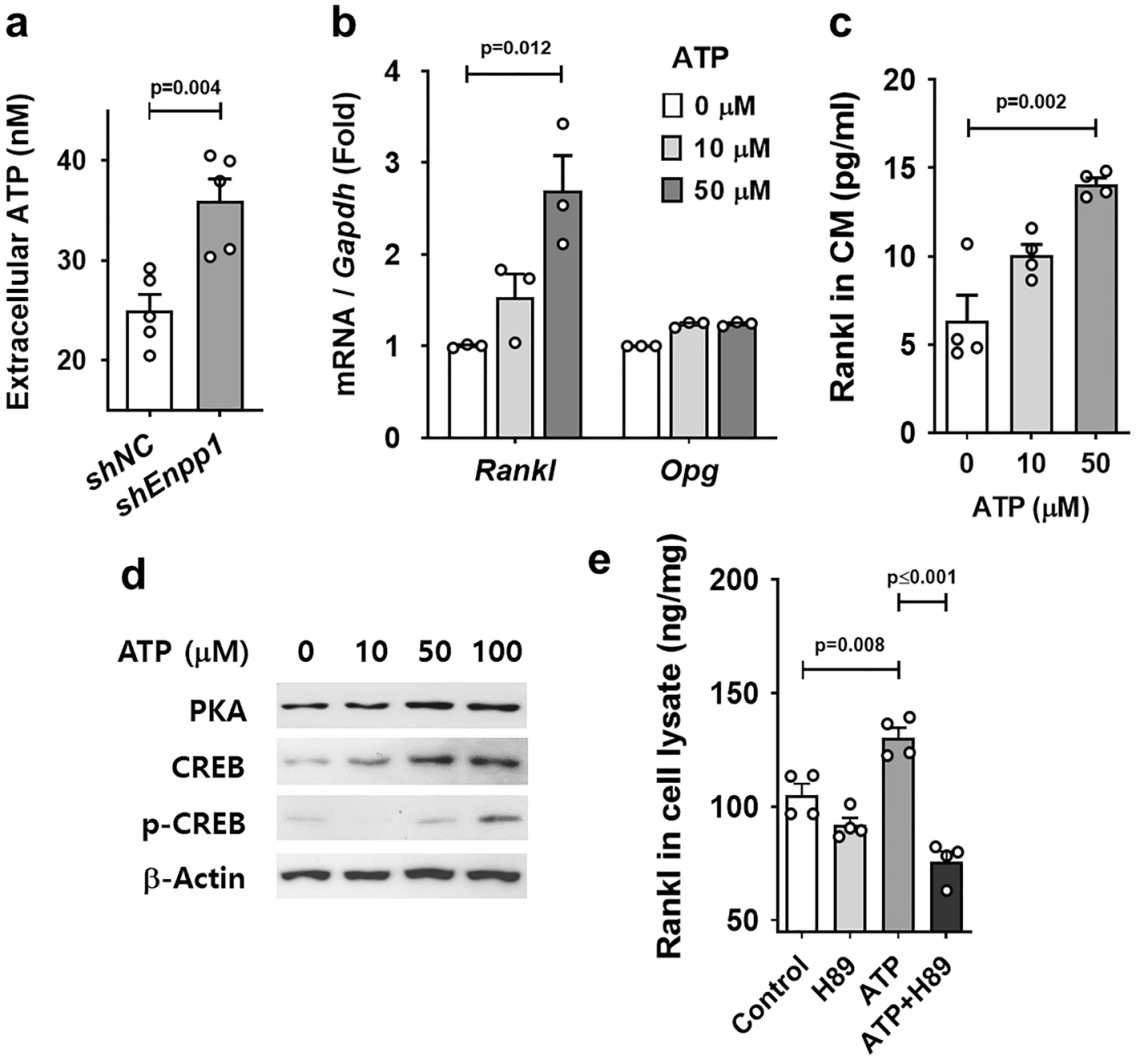
Extracellular ATP induces the expression of Rankl in cementoblasts. (**a**) Extracellular ATP was measured with conditioned media (CM) from the culture of OCCM-30 cells with *shEnpp1* and *shNC* (n = 5). (**b**) Transcript levels of *Rankl* and *Opg* were analyzed by real-time qPCR (n = 3). RNA was isolated from OCCM-30 cells treated with ATP for 24 h. (**c**) Protein amount of soluble Rankl was measured by ELISA using conditioned media (CM) from the culture of OCCM-30 cells treated with ATP (n = 4). (**d**) Protein levels were analyzed by Western blotting using specific antibodies with whole cell lysates from OCCM-30 cells treated with ATP for 24 h. Samples shown from Western blotting are from the same experiment, and the gels/blots were processed under the same experimental conditions. β-Actin was used as a loading control. Cropped images are displayed here; the original full-size blots are presented in Supplementary Fig. S6 online. (**e**) The protein amount of Rankl was measured by ELISA using whole cell lysates from OCCM-30 cells pretreated with H89 for 1 h and then treated with ATP (50 μM) for 48 h (n = 4). Significance was assigned with *p*-values as indicated in each graph.

Membrane-bound Npp1 preferentially hydrolyzes ATP to produce PPi with its catalytic domain located at the outer plasma membrane^21^. To investigate whether ATP is accumulated in the extracellular medium upon Npp1 inactivation, we analyzed the level of extracellular ATP using CM from the culture of OCCM-30 cells. The level of extracellular ATP was significantly increased in the CM of *Enpp1*-knockdown OCCM-30 cells compared with the control (Fig. 5a). Since extracellular ATP is a potent stimulator of osteoclastogenesis^22^, we sought the role of locally increased extracellular ATP responsible for the resorption of cementum in *Enpp1*^*asj*^ mice. The expression of P2 receptors specifically having known roles in the immune and inflammatory systems and bone remodeling system such as *P2X7*, *P2Y1*, *P2Y2*, and *P2Y6* was also analyzed in the *Enpp1*-knockdown OCCM-30 cells (Supplementary Fig. S3). The transcript level of these P2 receptors were slightly increased except *P2X7* by Enpp1 inactivation in OCCM-30. Since pericellular ATP can reach low micromolar range while the ATP concentration is in the low nanomolar range in the extracellular environment under physiological conditions^23^, the concentration range of ATP was adequately applied for in vitro experiments. The treatment of ATP significantly induced the expression of Rankl at both transcription and protein levels in OCCM-30 cells (Figs. 5b, c). To analyze the intracellular signaling of extracellular ATP in cementoblasts, Western blot analysis was performed with cell lysates from OCCM-30 cells treated with ATP. The protein levels of protein kinase A (PKA), cAMP-response element-binding protein (CREB), and its phosphorylated type (p-CREB) were increased in OCCM-30 cells treated with ATP in a concentration-dependent manner (Fig. 5d). As analyzed by ELISA, the induction of Rankl by ATP treatment was significantly impaired by the pretreatment of H89, an inhibitor of PKA (Fig. 5e). Taken together, these results suggest that extracellular ATP increased by *Enpp1* inactivation autocrinally induces Rankl expression through the PKA/CREB pathway in cementoblasts.

## Discussion

In this study, we observed that ectopically thickened cervical cementum caused by the functional inactivation of *Enpp1* is susceptible to resorption in vivo and investigated how Npp1 protects tooth roots through its PPi-generating function in physiological states of normal healthy teeth as a resorption barrier. In *Enpp1*^*asj*^ mice, the cervical cementum of the tooth is thickened by an ectopically accumulated matrix enriched with non-collagenous proteins including Bsp, Dmp1, and Opn^14^. Non-collagenous proteins in mineralizing tissues represent diverse entities capable of initiating and controlling the formation of hydroxyapatite crystals to regulate the process of bone mineralization in bone^17,24^. For example, Bsp and Dmp1 have been known to act as protein nucleators of hydroxyapatite^17,24^. Based on this, Npp1 is likely to play roles in the regulation of the local expression of these non-collagenous proteins as well as determining the quality and biomechanical properties of the produced mineralized matrix. The molecular composition of the thickened cervical cementum matrix of *Enpp1*^*asj*^ mice is likely similar with rapidly deposited and mineralized woven bone. Woven bone is more susceptible to osteoclastic resorption because of its RGD motif compared to lamellar bone^17,18^. Since the RGD sequence expressed in non-collagenous ECM molecules serves as a primary cell attachment cue, the molecular component of ECM affects cell adhesion. In the hard tissue catabolizing resorption process, osteoclast progenitor cells must colonize at the site of resorption. In this process, cell adhesion is required for the proper osteoclast differentiation of osteoclast precursor cells^25^. Non-collagenous protein components such as Bsp, Dmp1, and Opn bind to integrin αvβ3 of the osteoclast surface through its RGD sequence^26^. Through *Enpp1* inactivation, the differential expression of ECM proteins may act as a molecular signal for the selective adhesion of osteoclasts preferentially to ectopic cementum and its subsequent resorption^27,28^. Cementoblasts highly expressing Npp1 and Ank are recognized as highly pyrophosphate-sensitive cells. Maintenance of hard-soft interface at the tooth root surface depends on finely tuned PPi homeostasis in cementoblasts^10^. In this model of *Enpp1*^*asj*^, ectopic cementum formation seems to depend heavily on creation of a physicochemical environment conducive for apposition, such as by PPi clearance. It is likely that, while cementum and bone are made of similar extracellular matrix proteins, cementum is more vulnerable to resorption due to Npp1 deficiency than bone. Accordingly, the idea that there are key differences influencing acellular versus cellular cementum development is also supported. Namely, acellular cementum with high expression of Npp1 is dependent on precise modulation of local PPi, whereas cellular cementum is much less sensitive to local PPi^10^.

The control of non-collagenous proteins in ECM that consists of cervical cementum is likely important to preserve the integrity and biological properties of tissue. Our results demonstrated that β-catenin is one of the major regulators of ECM accumulation through Gsk3β that negatively conducts its activity. β-Catenin, the central target and an essential component of the Wnt/β-catenin signaling pathway, is involved in numerous aspects of growth and development in many organs and tissues, including osteogenic differentiation and function^29–31^. Overall, these studies indicate that canonical Wnt activation promotes osteogenesis through massive bone formation, while Wnt antagonists inhibit. Consistent with the results of bone, excessive cementum matrices were accumulated on the root surface in *osteocalcin* (*OC*)*-Cre:Catnb*^*lox/*+^ mice through the stabilization of β-catenin^19^. In our previous data, the treatment of PPi to OCCM-30 cells reduced the levels of non-phosphorylated active β-catenin and phosphorylated Gsk3α/β (p-Gsk3α/β; α^Ser21^ and β^Ser9^), indicating an increase in total Gsk3β activity and β-catenin degradation through Npp1 activity^14^. These results suggest that β-catenin activity appears to be suppressed by Npp1 through the regulation of PPi and Gsk3β in the physiological state^14^.

Compared with the role in the generation of PPi for anti-mineralization, the role of Npp1 in the regulation of extracellular ATP to contribute to ATP homeostasis has received little attention. As one of the primary enzymes responsible for ATP hydrolysis, Npp1 catabolizes extracellular ATP into AMP and PPi, implying that Npp1 concurrently serves as a metabolic regulator of ATP. Upon *Enpp1* inactivation, excessive extracellular ATP can be accumulated in the periodontium around the cervical cementum. Extracellular ATP is a pro-inflammatory agent interacting with purinoreceptors present on fibroblasts and osteoblasts and inducing the generation of Rankl that further activates osteoclastic alveolar bone resorption and bone loss in periodontitis^32^. In our in vitro results, extracellular ATP is accumulated through *Enpp1* inactivation, autocrinally induces the expression of Rankl through PKA/CREB activation in cementoblast-like cells and eventually stimulates the differentiation of osteoclast progenitors.

In the blood circulatory system, soluble nucleotide pyrophosphatases play a role in efficiently coupling between the release of ATP into cell surface microenvironments and its rapid metabolism with other ecto-ATPases^33,34^. To prevent the activation of purinergic receptors in physiologic resting conditions, concentrations of ATP in extracellular compartments are maintained within a narrow nanomolar range through the coordinated coupling of ATP release mechanisms and the metabolism of released ATP via various ectonucleotidases^35^. Npp1 in the cervical cementum may contribute to the local metabolism of extracellular ATP which stimulates purinergic signaling at certain circumstances of inflammation or trauma. Therefore, Npp1 is a highly effective enzyme with tightly coupled dual activities such as ATP-inactivating and PPi-generating pathways. Based on these findings, it is likely that the rates of intracellular ATP release and extracellular ATP hydrolysis are, at least in a certain way, balanced by Npp1 in the physiological state of cervical cementum.

Taken together, it is strongly suggested that external root resorption rarely occurs during homeostasis in cervical cementum in its integrity of an intrinsic layer with functionally active Npp1. Therefore, functional Npp1 preserves cervical cementum integrity and supports the anti-resorptive properties of tooth roots through ATP homeostasis (Supplementary Fig. S4). These findings may significantly expand our understanding of the role of Npp1 not limited in anti-mineralization, but in the regulation of the duration and magnitude of cementum homeostasis against external root resorption.

## Methods

### Mice

All the experimental procedures were approved by the Animal Welfare Committee of Jeonbuk National University. All methods were performed in accordance with the ARRIVE guidelines and in accordance with the relevant guidelines and regulations. All the mice were housed in a temperature-controlled environment with 12 h light/dark cycles. The heterozygous *Enpp1*^*asj*^ mice have been previously described^36^. At least three independent littermates were used for each experimental group (n ≥ 5/genotype, including males and females, age indicated in the figure).

### Scanning electron microscopy (SEM) and tartrate-resistant acid phosphatase (TRAP) staining

To characterize the surface structures of the tooth root, we extracted mandibular first molars at the indicated age and obtained images by SEM as described previously^37^. For TRAP staining, the paraffin sections were rehydrated and stained with a TRAP staining kit (387A, Sigma-Aldrich, St. Louis, MO, USA) according to the manufacturer’s instructions. The average TRAP-positive area was measured using the analySIS Pro imaging system (Soft Imaging System, Münster, Germany). For this calculation, three measurements from the representative slides in each group were used.

### Cell culture and treatment

OCCM-30, a mouse cementoblast cell line, was provided by Dr. Martha J. Somerman (National Institutes of Health, Bethesda, MD, USA) and cultured as described^38^. Stable cell lines with short hairpin RNA against mouse *Enpp1* (*shEnpp1*) or the negative control (*shNC*) were described previously^39^. To induce cell differentiation and mineralization, 95% confluent cells were cultured in the medium supplemented with 2% fetal bovine serum, 50 μg/ml ascorbic acid (Sigma Aldrich), and 10 mM β-glycerophosphate (Sigma Aldrich) for up to 4 days. Cells at 95% confluency were treated with adenosine triphosphate (ATP; A2383, Sigma Aldrich), Enpp1 inhibitor C (29809, Cayman, Ann Arbor, MI, USA), and H89 (ab120341, Abcam, Cambridge, MA, USA) as indicated. BMMs were obtained from femur bone marrow and were cultured in α-MEM (LM008-01, Welgene, Gyeongsan, South Korea) with 10% FBS (16000044, GIBCO, Waltham, MA, USA) containing 20 ng/ml recombinant human macrophage colony-stimulating factor (M-CSF; 300–25, Peprotech, Cranbury, NJ, USA). For osteoclastic differentiation, BMMs were suspended with growth media supplemented with 10 ng/ml recombinant mouse Rankl (462-TEC, R&D Systems) for 9 days. The plasmids driving the expression of mouse *β-catenin deltaGsk* (*β-CatΔGsk*), a gift from Tannishtha Reya (Addgene plasmids #14717), and human *GSK3β S9A* constitutively active mutant, a gift from Jim Woodgett (Addgene plasmids #14754), constructs were transfected as described^39^.

### Statistical analysis and supplementary information

Values in each graph represent mean ± standard error of the mean (SEM). Normal data with equal variance were analyzed using Student’s t-test, or the one-way analysis of variance (ANOVA) test. Significance was assigned for *p* ≤ 0.05. All assays were performed at least three times, with representative data presented. Detailed descriptions for other experimental materials and methods are provided in the Supplementary Methods.

## Supplementary Information

Supplementary Information.

## Data Availability

The datasets used and/or analyzed during the current study available from the corresponding author on reasonable request.
