# Supplementary material for: Npp1 prevents external tooth root resorption by regulation of cervical cementum integrity
Source: Sci Rep. 2022 Dec 7;12:21158. doi: 10.1038/s41598-022-25846-3 (PMC9729310; doi:10.1038/s41598-022-25846-3)
Supplement: Supplementary file 1 — Supplementary Information. [file 41598_2022_25846_MOESM1_ESM.pdf]

Supplementary Information

Npp1 prevents external tooth root resorption by regulation of cervical cementum integrity

Hwajung Choi, Liu Yang, Yudong Liu, Ju-Kyung Jeong, Eui-Sic Cho*

Cluster for Craniofacial Development and Regeneration Research, Institute of Oral Biosciences, Jeonbuk National University School of Dentistry, Jeonju 54896, South Korea

Supplementary Methods

Tissue preparation, immunohistochemistry (IHC) and histomorphometry

For histologic analysis, the dissected mandibles were fixed in 4% paraformaldehyde and decalcified in 10% EDTA for 4 weeks at 4°C. The decalcified tissues were dehydrated through a graded ethanol series, embedded in paraffin, and sectioned at 5-μm thickness. Slides were stained with H-E. For immunohistochemical staining, sections were treated with 3% hydrogen peroxide and incubated with rabbit polyclonal antibodies as described. The Histostain Plus Rabbit Primary DAB kit (Zymed Laboratories, San Francisco, CA, USA) were used following the manufacturers' instructions. Cervical cementum thickness was measured as described[1].

RNA preparation and real-time qPCR

Total RNA was prepared using AccuPrep® Universal RNA Extraction kit (K-3140, Bioneer, Daejeon, South Korea) according to the manufacturer's instructions, and cDNA was synthesized from 3 μg of total RNA using Superscript II reverse transcriptase (100004925, Invitrogen, Carlsbad, CA, USA). Real-time qPCR was performed using specific primer sets listed in Supplementary Table S1 as described previously[2].

Nucleoside triphosphate pyrophosphohydrolase (NTPPPHase) activity and quantitation of extracellular ATP

To measure the activity of total NTPPPHase, cells were incubated in phenol red-free medium for 2 hours with 1 mM thymidine 5' monophosphate p-nitrophenyl ester sodium (T4510, Sigma-Aldrich, St. Louis, MO, USA) solution at 37 °C. After incubation, 80 μl of supernatant for each condition was transferred to a 96 well plate containing 80 μl of 0.1 N NaOH. Absorbance was recorded at a wavelength of 410 nm. Extracellular ATP levels were

measured using ATP assay kit (ab83355, Abcam, Cambridge, MA, USA).

Western blot analysis and enzyme-linked immunosorbent assay (ELISA)

Western blot analysis was performed using specific antibodies against non-phosphorylated active β-catenin (8814, Cell Signaling), β-catenin (RB9035, Thermo Scientific), GSK-3β (9315, Cell Signaling), phosphor-GSK-3α/β (9331, Cell Signaling), protein kinase A (4782, Cell Signaling Technology, Danvers, MA, USA), CREB (sc-377154, Santa Cruz Biotechnology), phosphorylated-CREB (p-CREB; sc-81486, Santa Cruz Biotechnology) and β-actin (sc-1616R, Santa Cruz Biotechnology) as described previously[3]. For ELISA, the production of Rankl was measured in the whole cell lysate and conditioned media from the cell culture using the ELISA kit (LS-F5674, LSBio, Seattle, WA, USA) following the manufacturers' instructions.

Mineralization induction and alizarin red staining

Cells at 95% confluency were cultured in osteogenic medium supplemented with 2% fetal bovine serum, 50 μg/ml ascorbic acid (Sigma Aldrich), and 10 mM β-glycerophosphate (Sigma Aldrich), for 7 days. Mineral nodule formation was then observed by staining the cells with 40 mM alizarin red S (pH 4.2) after fixation with 4% PFA for 10 minutes. The amount of alizarin red S that bound to the minerals was quantified by destaining the samples in 10 mM sodium phosphate containing 10% cetylpyridinium chloride (pH 7.0) for 15 minutes at room temperature. The amount of alizarin red S in the destaining solution was measured at OD 562 nm.

Alkaline phosphatase (ALP) activity and staining

Cells were cultured in osteogenic medium for 48 hours. Alkaline phosphatase activity was quantitated using an assay based on the hydrolysis of p-nitrophenylphosphate (p-NPP) to p-nitrophenol (p-NP). Briefly, cell layers were washed twice with ice-cold PBS and lysed in 50 mM Tris–HCl buffer (pH 7.0) containing 1% (v/v) Triton X-100 (Sigma Aldrich) and 1 mM PMSF (Sigma Aldrich). Whole cell lysates were assayed by adding 1 mg/ml of pNPP substrate in 0.1 M glycine buffer (pH 10.4) containing 1 mM $ZnCl_2$ (Sigma Aldrich) and 1 mM $MgCl_2$ (Sigma Aldrich) to each tube for 15 min at 37 °C. Reactions were stopped by adding NaOH (final concentration 0.6 N), and the absorbance of each lysate was measured spectrophotometrically at 405 nm. Enzyme activity was normalized to total protein content and expressed using the fold change. For ALP staining, cells were stained with a Leukocyte Alkaline Phosphatase kit (Sigma Aldrich) according to the manufacturer's protocol.

Supplementary Table S1. Primer sequences for real-time qPCR

Gene	Sense	Antisense
Rankl	ATGAAAGGAGGGAGCACGAA	GTACGTCGCATCTTGATCCG
Opg	ACGGACAGCTGGCACACCAG	CTCACACACTCGGTTGTGGG
Bsp	AAAGTGAAGGAAAGCGACGA	GTTCCTTCTGCACCTGCTTC
Trap	AGACCCAGACCCTGAACACC	CGCCCAAGAAAGCTCTACCTAA
CtsK	CACGGCAAAGGCAGCTAAAT	CCATAGCCCACCACCAACAC
Dmp1	AGTGAGTCATCAGAAGAAAGTCAAGC	CTATACTGGCCTCTGTCGTAGCC
Opn	CCCGGTGAAAGTGACTGATTC	ATGGCTTTCATTGGAATTGC
P2X7	GGGTCTTGCACATGATCGTC	GGGAAGGTGTAGTCTGCAGT
P2Y1	CAATGTGCCCTGACCAAGAC	CTGGTAGGGTGAGCACGTAT
P2Y2	TCTTCCTATGCCGCCTCAAA	ATGCTGCAGTAGAGGTTGGT
P2Y6	GTATACTCGGTGGTGCTGGT	GTAGGGGTAGTGAACAGGCA
Gapdh	TGCCCAGAACATCATCCCT	GGTCCTCAGTGTAGCCCAAG

Legends for Supplementary Figures

Supplementary Figure S1. Lower magnified TRAP-staining images of Figure 1b. The TRAP-positive area of cervical cementum and faced alveolar bone were exhibited with tissue sections of the mandibular first molar at P28 (Top) and P56 (Bottom). The right images of each genotype are higher-magnification views of cementum and cementum-faced alveolar bone in the boxed area of each image. Black arrow heads indicate Npp1-positive and TRAP-negative cervical cementum of WT. Yellow arrow heads indicate resorption lacunae of the thickened cervical cementum layer of *Enpp1*^{asj} mice. In *Enpp1*^{asj} mice, most of the lacunae are found preferentially on the distal root surface of molars. Since teeth drift distally, most of the lacunae

were found preferentially on the cementum-faced mesial sides of alveolar bone (blue arrow heads) while distal sides of alveolar bone were TRAP-negative (white arrow heads) in both mice. D, dentin; PDL, periodontal ligament; C, cementum; AB, alveolar bone. Scale bars are indicated.

Supplementary Figure S2. Npp1 inhibits mineralization of cementoblasts *in vitro*. ALP activity with ALP staining and mineralization ability by Alizarin red S staining were analyzed with OCCM-30 cells with *shEnpp1* and *shNC* differentiatied for 7 days.

Supplementary Figure S3. Inactivation of *Enpp1* slightly induces the expression of P2 receptors in cementoblasts. Transcript levels of *P2X7*, *P2Y1*, *P2Y2* and *P2Y6* were analyzed by real-time qPCR (n = 3). RNA was isolated from OCCM-30 cells with *shEnpp1* and *shNC*. Significance was assigned with *p*-values as indicated in the graph.

Supplementary Figure S4. Functional Npp1 preserves anti-resorptive cervical cementum by regulation of cervical cementum integrity. (a) In physiological state, the PPi-generating enzyme activity of Npp1 using ATP is important for preventing hydroxyapatite (HA) crystal formation on the surface of cervical cementum. Through inorganic pyrophosphate (PPi), Npp1 suppresses β-catenin activity that regulates non-collagenous matrix apposition. Hence, functional Npp1 preserves thin and less-mineralized cementum integrity and supports anti-resorptive property of cervical cementum through ATP homeostasis. (b) Disruption of Npp1 function leads to shortage of PPi and consequently induces HA formation with higher Tnap activity. In addition, the shortage of PPi induces β-catenin stabilization and results in RGD-rich matrix accumulation on the surface of cervical cementum. This ectopic formation of cementum is susceptible to resorption by increased expression of Rankl through redundant

extracellular ATP.

Supplementary Figure S5. Original full-size blots of Figure 3a. The following antibodies were used: non-phosphorylated active β-catenin (Act. β-Cat, 1:2000), β-catenin (β-Cat, 1:2000), Gsk3β (1:1000), phosphor-Gsk-3α/β (p-GSK3α/β, 1:1000) and β-Actin (1:2000). The full-length blots had been occasionally cut before hybridization with antibodies, generating shorter length of images.

Supplementary Figure S6. Original full-size blots of Figure 5d. The following antibodies were used: Protein kinase A (PKA, 1:1000), CREB (1:500), phosphorylated-CREB (p-CREB, 1:200) and β-Actin (1:2000). Asterisks indicate that other experimental samples were simultaneously analyzed with ATP-treated OCCM-30 WCL samples sharing the control for convenience.

References

1 Choi, H. *et al.* A Reciprocal Interaction between beta-Catenin and Osterix in Cementogenesis. *Sci Rep* **7**, 8160, doi:10.1038/s41598-017-08607-5 (2017).

2 Choi, H. *et al.* TGF-beta Signaling Regulates Cementum Formation through Osterix Expression. *Sci Rep* **6**, 26046, doi:10.1038/srep26046 (2016).

3 Choi, H., Liu, Y., Jeong, J. K., Kim, T. H. & Cho, E. S. Antagonistic interactions between osterix and pyrophosphate during cementum formation. *Bone* **125**, 8-15, doi:10.1016/j.bone.2019.05.001 (2019).

Supplementary Figure S1

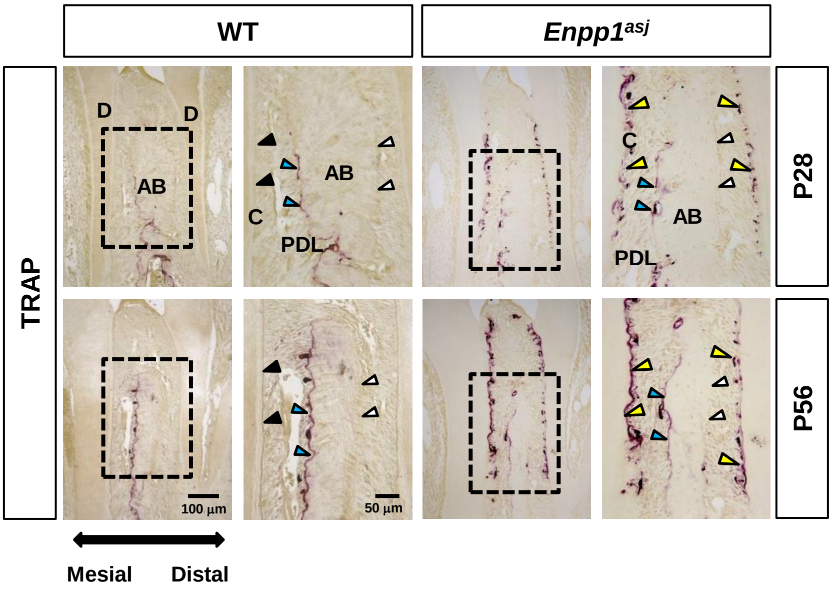

Supplementary Figure S2

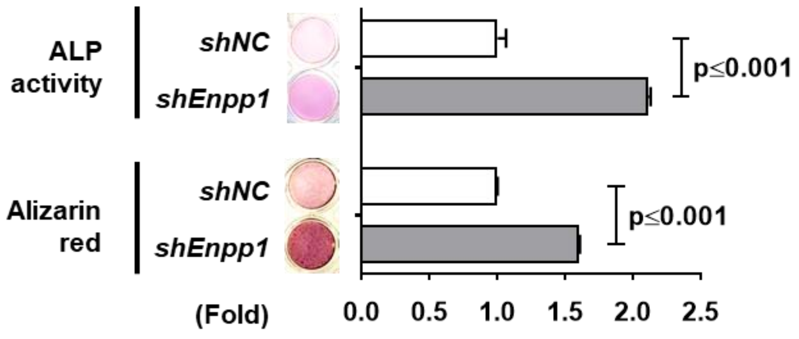

Supplementary Figure S3

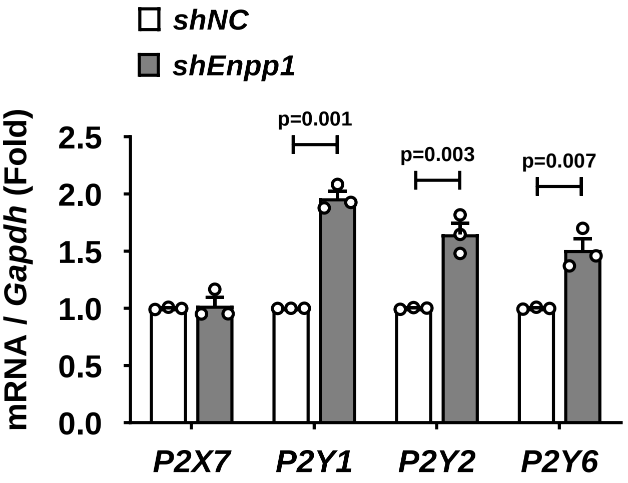

Supplementary Figure S4

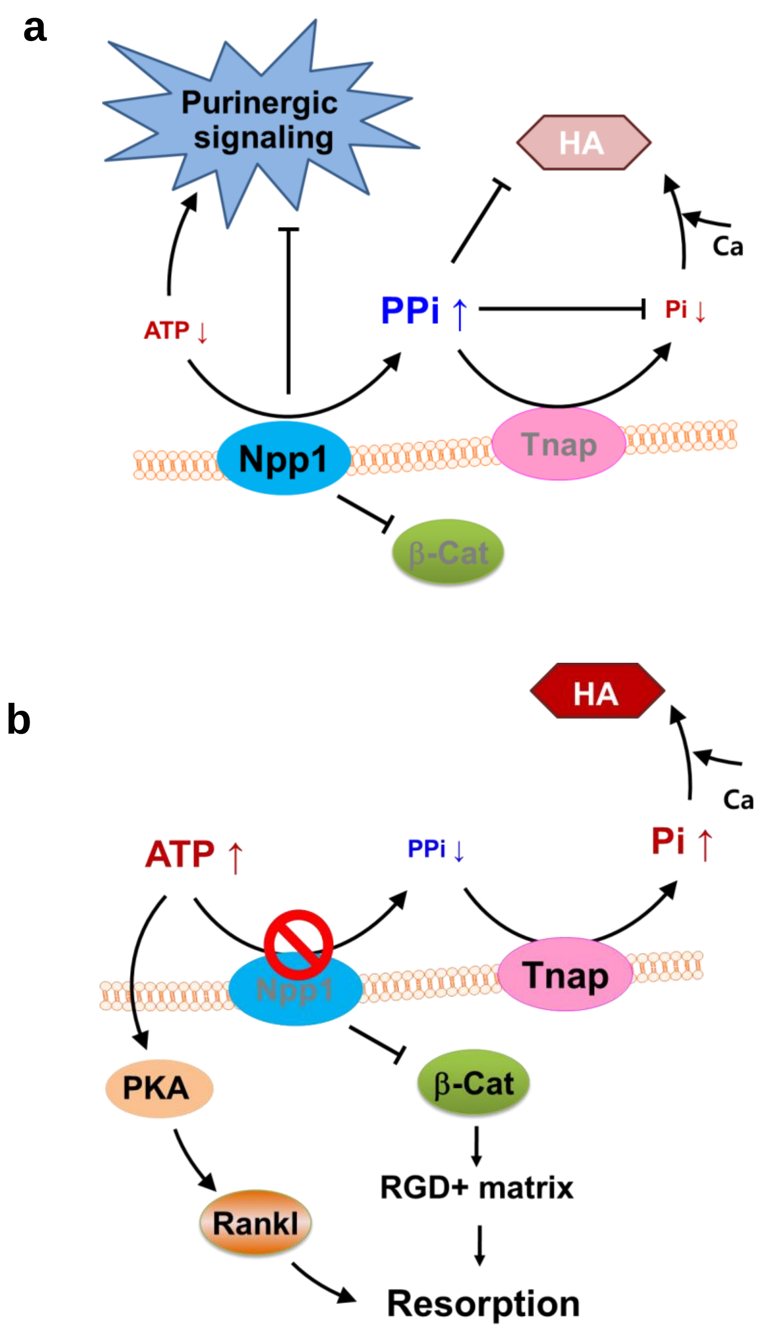

Supplementary Figure S5

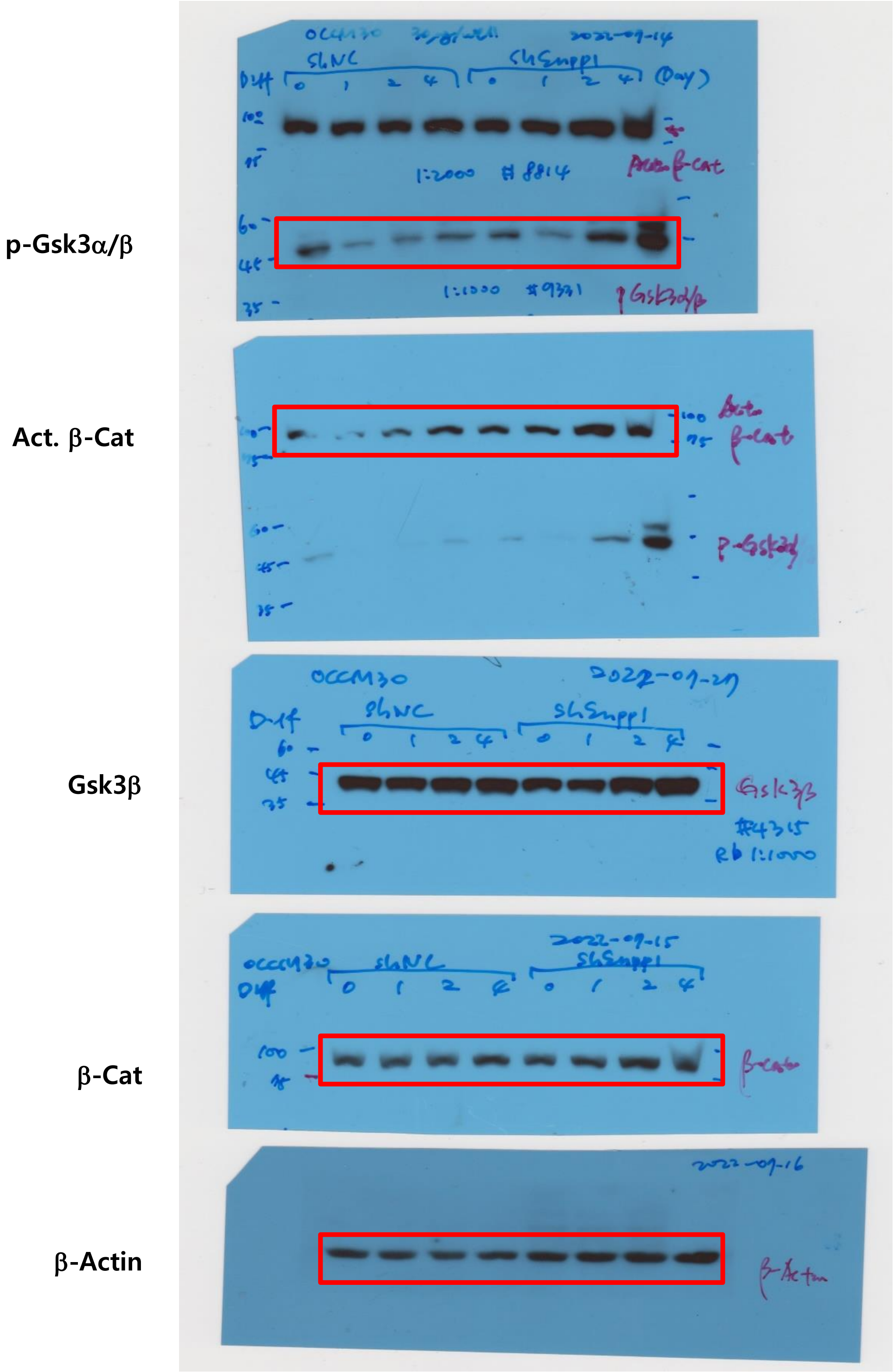

Supplementary Figure S6

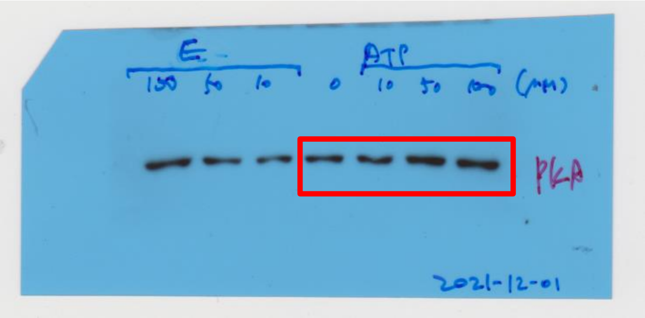

PKA*

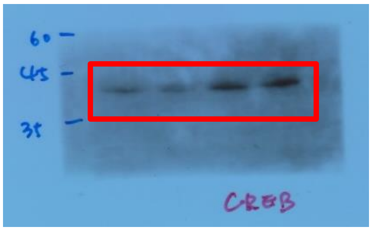

CREB

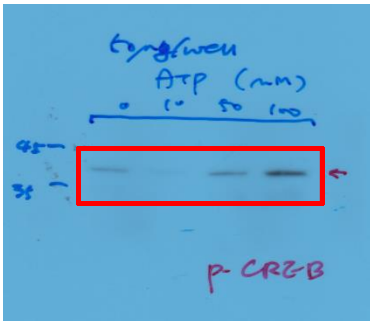

p-CREB

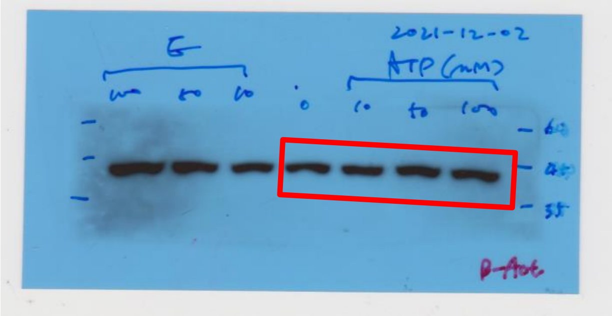

β-Actin*
